# When Menstrual Pain Becomes Care‐Worthy: A Qualitative Study of Delayed Care‐Seeking Among Young Women With Function‐Limiting Dysmenorrhoea in Southwest China

**DOI:** 10.1111/hex.70930

**Published:** 2026-10-23

**Authors:** Qingqing Zhu, Bentao Jia, Qi Yu, Mingqiang Wang, Wei Xie, Ling Qi, Hongbo Chen, Min Wang, Xiping Li

**Affiliations:** ^1^ Guizhou University of Traditional Chinese Medicine Guiyang China; ^2^ The Affiliated Hospital of Guizhou Medical University Guiyang China; ^3^ Guizhou Nursing Vocational College, Qiannan Buyi and Miao Autonomous Prefecture Guizhou China

**Keywords:** delayed care‐seeking, dysmenorrhoea, menstrual health, qualitative research, Southwest China, traditional Chinese medicine, young women

## Abstract

**Background:**

Dysmenorrhoea can disrupt education, work, sleep, mobility and other daily activities, yet recurrent pain that limits daily functioning does not always prompt timely formal care‐seeking.

**Objective:**

The aim of this study was to explore how young women in Southwest China interpret recurrent function‐limiting dysmenorrhoea and decide whether to seek formal care.

**Methods:**

This qualitative study was based in Guiyang, Guizhou Province, Southwest China. Seventeen women aged 18–31 years with recurrent dysmenorrhoea affecting daily functioning took part in semi‐structured interviews. Interviews were conducted face‐to‐face or online, audio‐recorded, transcribed verbatim and analysed using reflexive thematic analysis.

**Results:**

Five themes were developed: making pain ordinary across family, peers and clinics; keeping pain within the logic of warmth and regulation; when pain becomes care‐worthy: functional disruption as a threshold; reproductive expectations and delayed medicalisation; and normal tests, uncertain explanations and delayed re‐entry into care. Care‐seeking was often delayed as participants normalised menstrual pain, relied on culturally familiar warming or body‐regulating practices, had concerns about analgesics or expected pain to improve after childbirth. Non‐specific clinical explanations could also temporarily reassure participants and contribute to delay. Participants described seeking formal care when pain disrupted study, work, sleep, mobility or daily activities, when medication or self‐management no longer worked, or when reproductive or gynaecological concerns emerged.

**Conclusions:**

Delayed care‐seeking was shaped by whether menstrual pain became recognised as a legitimate reason for formal care. Menstrual health education and clinical communication should validate function‐limiting pain, clarify when assessment is needed and provide clear follow‐up guidance.

**Patient or Public Contribution:**

Four women with lived experience of function‐limiting dysmenorrhoea helped develop parts of the interview guide. Their feedback during pilot interviews led to additional questions about healthcare access and practical barriers to seeking care. They also supported recruitment and contributed to discussions of interview accounts and developing themes.

## 1 Introduction

Dysmenorrhoea is one of the most common menstrual health problems among adolescents and women of reproductive age. It affects around 71% of menstruating women and girls globally [1]. Dysmenorrhoea has important physical, psychological and social consequences. When menstrual pain limits daily functioning, it can interfere with education, work, sleep, mobility, social participation and quality of life, and is consistently associated with absenteeism and impaired daily activities [2, 3]. Reflecting this burden, the World Health Organization identifies menstrual pain and other menstrual symptoms that disrupt normal activities as valid reasons for clinical evaluation [4]. Despite this, function‐limiting menstrual pain does not always lead to timely formal care [5, 6].

The burden of dysmenorrhoea is also evident in China. A cross‐sectional survey of women attending a hospital in Nanjing identified dysmenorrhoea as the most commonly reported gynaecological condition in that sample [7]. A study of junior high school girls in Shanghai linked academic stress to dysmenorrhoea and school absenteeism and examined factors associated with healthcare use [8]. Decisions about seeking care are made within everyday social and healthcare contexts, in which family members, peers, teachers and healthcare providers shape whether menstrual pain is regarded as normal, manageable, serious or requiring medical attention [9, 10].

Menstrual literacy refers to the cognitive and social skills needed to understand, use and communicate menstrual health information to support menstrual and general wellbeing [11]. It shapes how young women interpret dysmenorrhoea, manage symptoms and decide whether clinical assessment is needed. However, familiarity with menstruation does not necessarily translate into knowing how to recognise abnormal symptoms, manage pain safely or judge when clinical assessment is indicated [12]. Qualitative evidence further shows that menstrual experiences are shaped by access to information, social support, stigma and broader social and structural conditions [13]. When menstrual education does not address symptom appraisal, safe management and care‐seeking thresholds, young women may remain uncertain about when self‐management is appropriate and when function‐limiting pain warrants professional care.

Previous healthcare encounters may also complicate care‐seeking decisions. Although primary dysmenorrhoea is common and often responds to first‐line treatment, dysmenorrhoea may also indicate secondary conditions such as endometriosis or adenomyosis. Clinical reviews emphasise history‐taking, examination where appropriate, assessment of treatment response and evaluation for secondary causes when symptoms are severe, progressive or refractory [14, 15]. For young women, normal test results or brief reassurance may not resolve uncertainty if pain persists. Without clear explanations or follow‐up guidance, they may be unsure whether to return to care, seek another provider or continue self‐management.

Alongside menstrual literacy and experiences of clinical contact, culturally familiar explanations may shape how menstrual pain is understood and managed in China. Biomedical, traditional Chinese medicine and everyday family explanations coexist. Traditional Chinese medicine interprets dysmenorrhoea through pattern differentiation, including cold coagulation and blood stasis, qi stagnation and blood stasis, and qi and blood deficiency [16]. Within this framework, qi refers to the body's functional activity and capacity for movement, whereas the concept of blood encompasses nourishing and circulating functions and is not restricted to its biomedical meaning. Pain may therefore be attributed to a deficiency of qi or blood, or to obstruction of their movement by stagnation or cold [17]. Gong han, commonly translated as ‘cold uterus’, is a culturally familiar term shaped by both traditional Chinese medicine and everyday family beliefs. It does not denote a discrete biomedical diagnosis or a single standardised traditional Chinese medicine pattern. Instead, it links menstrual or reproductive discomfort with perceived internal cold and makes practices such as applying heat, drinking warm fluids and avoiding cold foods appear appropriate.

These practices are common among Chinese adolescents and young women. Advice about their use is often shared by mothers or peers, while professional consultation remains relatively uncommon [18, 19]. Menstruation may also be treated as a private matter. Expectations of concealment and endurance may make it difficult for young women to disclose pain, request leave from study or work, or regard functional disruption as a legitimate reason for seeking care [20, 21]. Together, culturally familiar self‐management and the normalisation of menstrual pain as an ordinary part of womanhood may raise the threshold at which recurrent or function‐limiting pain is judged to require formal care.

Although these beliefs and practices have been documented in China [18, 19, 20, 21], less is known about how young women negotiate them alongside healthcare encounters over time, particularly in Southwest China. Healthcare candidacy provides a conceptual lens for examining how function‐limiting pain becomes socially and clinically recognised as warranting formal care. It conceptualises access as a dynamic process through which individuals identify a need for care, navigate and present to services, and have their claims adjudicated by healthcare professionals within particular social and healthcare contexts [22, 23]. Applied to dysmenorrhoea, it directs attention to how family and peer normalisation, menstrual privacy, culturally familiar explanations, functional disruption and healthcare encounters shape entry into and movement through formal care.

This study aimed to explore how young women in Southwest China interpreted and responded to recurrent function‐limiting dysmenorrhoea. It examined how menstrual literacy, culturally familiar explanations, family and reproductive contexts, and healthcare encounters shaped delayed entry into, disengagement from and re‐entry into formal care.

## 2 Methods

### 2.1 Study Context and Setting

This study was based in Guiyang, the capital of Guizhou Province in Southwest China. Guizhou is a mountainous inland province. In 2024, 43.4% of residents lived in rural areas [24], and ethnic minority groups accounted for 36.4% of the population in the 2020 census [25]. Services accounted for 55.6% of provincial gross domestic product (GDP) in 2024, followed by industry and construction (31.3%) and agriculture and related activities (13.1%) [24]. GDP per capita was CNY 58,685, compared with CNY 95,749 nationally [24, 26].

Healthcare in Guizhou is provided through community health centres in urban areas, township health centres and village clinics in rural areas, and hospitals at county, municipal and provincial levels [27]. Biomedical and traditional Chinese medicine services coexist within this system. Studies in rural Guizhou have documented variation in the range of services offered by primary care facilities [28], as well as heavy workloads and poor working conditions among village doctors [29]. Patients can also seek care directly at higher‐level hospitals without a referral from primary care [27].

### 2.2 Study Design

This qualitative interview study examined how young women with recurrent function‐limiting dysmenorrhoea in Southwest China interpreted menstrual pain, managed their symptoms and made decisions about entering, continuing or re‐entering formal care. The study was reported in accordance with the Consolidated Criteria for Reporting Qualitative Research (COREQ) [30].

Ethical approval was granted by the Ethics Committee of the First Affiliated Hospital of Guizhou University of Traditional Chinese Medicine (Approval No. KY2024‐257A). All participants received information about the study and provided written or electronic informed consent before participation. Interviews were recorded with permission, transcripts were anonymised before analysis, and study materials were stored securely in accordance with institutional data governance requirements.

The multidisciplinary research team included members with backgrounds in nursing, women's health, traditional Chinese medicine, public health and qualitative research. These perspectives informed the interview guide and the interpretation of culturally embedded explanations of menstrual pain, self‐management and care‐seeking. Reflexive notes, analytic memos and team discussions were used to examine how the researchers’ professional backgrounds and assumptions shaped the developing analysis.

### 2.3 Recruitment and Data Collection

Women were recruited through university and community settings in Guiyang and through the authors’ social networks. The authors used purposive maximum‐variation sampling to include students and working women, women who had and had not given birth, and women with different experiences of seeking care for dysmenorrhoea. Age, educational background, ethnicity, marital status and rural or town/county background were also considered during recruitment. Seventeen women participated: eight students and nine working women. Some participants had previously worked outside Guizhou. Seven had never consulted a healthcare professional specifically for menstrual pain, while ten had sought such care. Those who had sought care reported attending community health centres, county‐level hospitals, traditional Chinese medicine hospitals and tertiary hospitals. Participant characteristics, including the occupations of the working women, are presented in Table 1.

**Table 1:** Participant characteristics (*N* = 17).

Characteristic	*n* (%), mean (SD), or median (IQR)
**Sociodemographic characteristics**	
Age, years	24.6 (3.7)
**Ethnicity**	
Han	12 (70.6)
Miao	4 (23.5)
Bai	1 (5.9)
**Residence background**	
Rural	15 (88.2)
Town or county seat	2 (11.8)
**Educational level**	
Secondary education or below	3 (17.6)
College or undergraduate	11 (64.7)
Postgraduate	3 (17.6)
**Current status**	
Student	8 (47.1)
Employed	9 (52.9)
**Occupation among employed participants (n** = **9)**	
Factory worker	1 (11.1)
Engineer	1 (11.1)
Construction‐sector technician	1 (11.1)
Nurse	1 (11.1)
Sales worker	2 (22.2)
Freelance worker	1 (11.1)
Clerical worker	1 (11.1)
Retail cashier	1 (11.1)
**Marital status**	
Unmarried	11 (64.7)
Married	6 (35.3)
**Parity**	
Nulliparous	14 (82.4)
Parous	3 (17.6)
**Menstrual and care‐seeking characteristics**	
Age at menarche, years	13 (13–14)
Age at dysmenorrhoea onset, years	15 (14–16)
**Care‐seeking trajectory**	
No clinical care sought specifically for menstrual pain	7 (41.2)
Healthcare contact without a participant‐reported diagnosis explaining the pain	9 (52.9)
Participant‐reported specialist diagnosis after repeated healthcare contact	1 (5.9)

Abbreviations: IQR, interquartile range; SD, standard deviation.

Women were eligible if they were aged 18 years or older, had menstruated for at least 1 year, and reported recurrent dysmenorrhoea affecting study, work, sleep, mobility or other daily activities. Eligibility did not depend on previous healthcare use, allowing women with different care‐seeking experiences to participate. Women unable to provide informed consent or complete an interview were excluded.

In this study, formal care referred to consultation with a healthcare professional in a recognised biomedical or traditional Chinese medicine setting. Self‐initiated use of analgesics, Chinese patent medicines, warmth‐based practices or family remedies without such consultation was treated as self‐management. Purchasing medicines from a pharmacy without consulting a healthcare professional was not classified as formal care.

The authors developed the topic guide with reference to previous research on menstrual pain, self‐management and care‐seeking [18, 19] and methodological guidance on semi‐structured interview guide development [31]. Four women with lived experience of function‐limiting dysmenorrhoea took part in pilot interviews and helped develop parts of the guide. Their feedback led to the addition of questions about access to healthcare and practical barriers to seeking care. They also supported recruitment by sharing study information or introducing potential participants. These women were not included in the main study sample.

Individual semi‐structured interviews were conducted by two researchers trained in qualitative interviewing. Interviews took place either face‐to‐face in a private setting or online via Tencent Meeting, a videoconferencing platform, according to participant preference. The guide explored participants’ interpretations of menstrual pain, symptom management, effects on daily life, family and peer responses, and experiences of seeking or not seeking formal care. Follow‐up questions explored issues raised by participants. Interviews lasted 30–90 min and were audio‐recorded with participants’ permission. Brief field notes were made after each interview.

Recruitment and preliminary analysis proceeded alongside data collection, with early interpretations informing subsequent interview questions. In later interviews, participants described explanations for menstrual pain, self‐management practices and reasons for seeking or not seeking care that were similar to those reported in earlier interviews. Following discussion, the authors judged that the interviews provided sufficient detail to examine how women understood and managed menstrual pain and decided whether to seek care. Recruitment therefore ended after 17 interviews.

### 2.4 Data Analysis

Interview recordings were transcribed verbatim in Mandarin Chinese, and the transcripts were checked against the recordings for accuracy. The authors analysed the data primarily in Mandarin using Braun and Clarke's six‐phase approach to reflexive thematic analysis [32, 33, 34, 35]. The analysis was predominantly inductive and experiential, focusing on participants’ descriptions and interpretations of menstrual pain, self‐management and care‐seeking.

Two of the authors who had conducted the interviews led familiarisation, coding and initial theme development. They read and re‐read the transcripts, recorded initial analytic observations and generated codes across the dataset. NVivo (version 15; Lumivero) was used to support data organisation and coding [36]. Coding addressed pain appraisal, symptom explanations, self‐management, disruption to daily life, reproductive expectations, healthcare encounters and decisions about seeking, discontinuing or returning to care. The authors examined related codes and the corresponding interview extracts to develop candidate themes. Each theme captured a pattern of shared meaning organised around a central concept. The authors reviewed these themes against the coded extracts and the full dataset, moving between codes and themes to refine each theme's focus, boundaries and name. Writing the findings contributed to this refinement.

During coding and theme development, the authors compared accounts from students and working women, focusing on how they discussed menstrual pain, requested leave and managed study or work responsibilities. Marital and reproductive circumstances and previous healthcare use were considered when interpreting participants’ reasons for seeking care, returning to self‐management or seeking further assessment. Ethnicity and rural or town/county background were considered when interpreting individual accounts; their uneven representation limited comparisons between groups.

After developing and refining the candidate themes, the authors used the healthcare candidacy framework to interpret the findings. The framework was selected because it considers how individuals and health professionals judge whether symptoms warrant care, and how these judgements are negotiated and reconsidered over time [22, 23]. This was relevant to understanding why function‐limiting menstrual pain did not always prompt consultation and how previous clinical encounters influenced decisions about further care. The analysis examined how family and peer advice and experiences in educational and workplace settings shaped participants’ views of whether pain warranted care, and how clinical explanations informed continued self‐management or further consultation.

Post‐interview notes, reflexive and analytic memos, coding notes and successive versions of the themes documented the analytic process. Three team members reviewed transcripts, coded extracts and developing interpretations; two additional members reviewed the broader analytic materials; and two senior qualitative researchers provided methodological supervision. Regular discussions examined the authors’ assumptions, alternative interpretations and accounts that challenged the developing themes. Women who had helped develop the interview guide also contributed to discussions of interview accounts and developing themes with the research team.

Selected quotations and key analytic terms were translated into English and checked by bilingual members of the research team. Translation prioritised conceptual and contextual equivalence. Minor language editing improved readability without changing participants’ intended meanings. Ellipses indicate omissions from quotations.

## 3 Results

Five interrelated themes were developed: making pain ordinary across family, peers and clinics; keeping pain within the logic of warmth and regulation; when pain becomes care‐worthy: functional disruption as a threshold; reproductive expectations and delayed medicalisation; and normal tests, uncertain explanations and delayed re‐entry into care. Together, these themes explain how young women negotiated whether recurrent function‐limiting dysmenorrhoea warranted formal care. Illustrative quotations are presented within each theme, and the relationships between the themes and the resulting care‐seeking trajectory are summarised in Figure 1.

**Figure 1:**
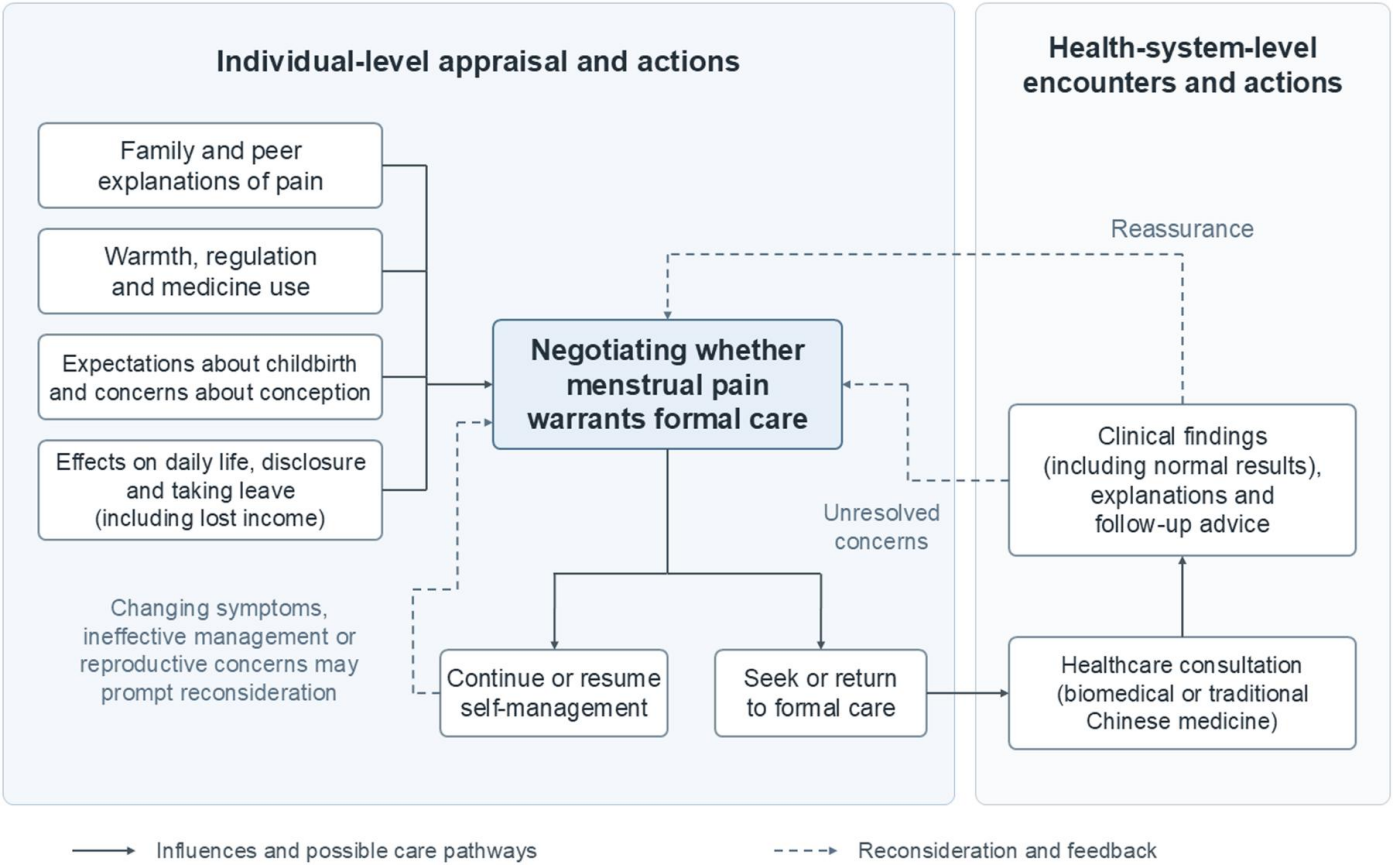
Negotiating healthcare candidacy across dysmenorrhoea care‐seeking trajectories.

### 3.1 Theme 1. Making Pain Ordinary Across Family, Peers and Clinics

Family members, particularly mothers, were the main source of participants’ initial explanations for menstrual pain. Mothers commonly drew on their own experiences to describe pain as a normal part of being female and something to be endured or managed through familiar self‐care practices. These messages reassured participants and shaped whether they considered medical attention necessary. P001 recalled:

My mother said it was normal, that girls are all like this and should just endure it. She said that she also had pain when she was young, and it became better after she gave birth to me. … She also said it would get better when I grew up and married. So I did not worry much. I just thought it was normal.

(P001, student)

The influence of family advice was strengthened by trust and shared experiences of pain. P002 described accepting her mother's explanation because she trusted her judgement, while her sister's improvement after childbirth gave that explanation further credibility. P007 similarly linked her understanding of pain as normal to messages from her mother and sister. Family experiences therefore provided both explanations for pain and examples that made endurance or self‐management appear appropriate.

Comparisons with classmates, friends and co‐workers further reinforced these family explanations. Participants judged their own symptoms against the pain that other women described or appeared able to tolerate. P005 explained:

If I were the only one with dysmenorrhoea, I would think I was ill and I would go to see a doctor. But most people have period pain, so it becomes something very normal. If it is not unbearable, I will not go.

(P005, construction‐sector technician)

Because P005 regarded menstrual pain as common, she considered consultation necessary only when it became unbearable. P007 also described colleagues managing pain with medication without considering further assessment, reinforcing the view that menstrual pain could be managed without consultation.

Among participants who had sought care, clinical explanations could echo messages already received within the family. P013 recalled being told that examination findings were normal and receiving advice to drink hot water and rest:

Every time after the examination, they said the results were normal, that there was no organic problem, and that dysmenorrhoea was normal. They told me to drink more hot water and rest more.

(P013, self‐employed)

P004 likewise described the advice she received from a doctor as similar to her mother's reassurance. These accounts show how clinical advice could repeat family messages that menstrual pain was normal. P013 nevertheless remained concerned about the cause of her pain and sought assessment at other hospitals.

Peer interactions could also encourage consultation or introduce alternative explanations for pain. P004 described colleagues urging her to seek help when she appeared unwell at work. P016 recalled first hearing about endometriosis through a co‐worker:

A co‐worker told me that her cousin also had severe period pain and was later diagnosed with endometriosis. That was the first time I had heard of it.

(P016, administrative worker)

Family advice thus shaped initial interpretations of pain, while peer comparisons and clinical explanations could reinforce them. Other peer interactions encouraged consultation or introduced the possibility that severe menstrual pain might have a different explanation.

### 3.2 Theme 2. Keeping Pain Within the Logic of Warmth and Regulation

Participants described managing dysmenorrhoea through warmth‐based and body‐regulating practices, including drinking hot water, brown sugar water or ginger tea; applying heat; soaking the feet; and avoiding cold foods and drinks. They also used herbal preparations, ‘womb‐warming’ patches and Chinese patent medicines, while some consulted traditional Chinese medicine practitioners. These responses were connected to explanations involving coldness, bodily weakness, insufficient qi and blood, obstructed flow or a body requiring regulation. Participants drew on family knowledge, everyday bodily observations, traditional Chinese medicine concepts and biomedical language when interpreting and managing pain.

Warmth was a familiar response commonly recommended by mothers or older female relatives and could be used privately at home, school or work. P016, who reported having received a diagnosis of endometriosis after laparoscopy, described how family advice directed her towards warming practices rather than clinical assessment:

My mother told me to drink brown sugar ginger tea, use heat patches, and not eat cold things. She never said I should go to see a doctor. She thought seeing a doctor would not help, because it was not really an illness.

(P016, administrative worker; participant‐reported diagnosis of endometriosis)

For some participants, explanations involving coldness drew on associations between pain, the season, exposure to cold and food. P012 recalled that her first episode of severe menstrual pain occurred during winter; both she and her family attributed it to bodily weakness or exposure to cold:

It was winter when I first menstruated, and the pain was very severe. I thought that perhaps my body was not very good and that I was relatively weak. My parents, especially my mother, thought that I might have become cold, or that my body was weak.

(P012, sales worker)

P012 used hot water, foot soaking, heat and moxibustion and later sought traditional Chinese medicine treatment. She reported temporary improvement after this treatment, followed by recurrence in winter that she associated with exposure to cold. She also recalled episodes of pain after eating ice lollies during the summer. These experiences reinforced her understanding of pain in relation to coldness and bodily weakness.

Different explanations could coexist within the same account. P009 described repeated consultations and used the term prostaglandins when considering a possible explanation for her pain:

I think all I can do is take medicine and avoid cold foods during my period. I have seen doctors many times, and they always tell me it is primary dysmenorrhoea. There does not seem to be much else I can do. Maybe I am particularly sensitive to prostaglandins.

(P009, student)

P009 suggested that sensitivity to prostaglandins might explain her pain, while also emphasising the need to avoid cold foods. In her account, a biomedical explanation of pain coexisted with the belief that cold foods could worsen symptoms.

P011 described how a traditional Chinese medicine practitioner explained and assessed her symptoms in terms of insufficient qi and blood:

The doctor felt my pulse and looked at my tongue. … He said I had insufficient qi and blood. … I still feel that my qi and blood are insufficient.

(P011, student)

P011 recalled that the practitioner had described her as weak and prescribed herbal medicine. Her mother also prepared soups intended to nourish qi and blood. At the time of the interview, P011 had not yet noticed an improvement and continued to describe herself as having insufficient qi and blood. This explanation informed both her understanding of pain and the treatment and care she received from the practitioner and her mother.

Chinese patent medicines, which are ready‐made Chinese medicinal formulations, were another form of management. P015 initially used Yuanhu Zhitong tablets purchased by her family:

When the pain was very severe, I would take medicine. At first, I took a Chinese patent medicine. My family bought it from a pharmacist at a pharmacy they knew. I remember it was Yuanhu Zhitong tablets.

(P015, sales worker)

P015 later sought traditional Chinese medicine care after changes in her symptoms: pain began before menstruation, and bleeding became scant but prolonged. The practitioner initially prescribed Chinese patent medicines and later switched to a herbal decoction when her symptoms did not improve. Her account shows that Chinese patent medicines could be used in both family‐initiated self‐management and practitioner‐guided treatment. Treatment with the decoction later stopped because her family feared that prolonged use might harm her kidneys.

Concerns about analgesics further supported reliance on warmth and regulation. Participants described fears of side effects, dependence, addiction, gastric injury or interference with menstrual discharge. P013 explained:

I usually do not take painkillers. People say they create dependence. If you take them the first time, then every time you have your period you have to take them for the pain to stop.

(P013, self‐employed)

Participants used warming practices and medicines to relieve pain and address what they understood as its underlying cause. Beliefs about warmth and bodily regulation informed both self‐management and practitioner‐guided treatment. Temporary relief could make further assessment seem less necessary, while persistent or changing symptoms prompted some participants to seek care or try a different treatment.

### 3.3 Theme 3. When Pain Becomes Care‐Worthy: Functional Disruption As a Threshold

Pain that interfered with study, work or other daily activities could prompt participants to consider formal care. However, disruption did not always lead to consultation. Participants’ accounts also showed how concerns about discussing menstruation and requesting leave shaped the way they managed pain at school or work.

P005 distinguished between pain that could still be endured and pain that justified consultation:

The standard is when the pain makes me unable to work or do things normally. If I can still hold on, I will not go.

(P005, construction‐sector technician)

Even when pain disrupted daily functioning, disclosing menstruation and requesting leave could remain difficult. P001 stayed in class despite being unable to concentrate because requesting leave would have required her to discuss menstruation with a male teacher:

I did not ask for leave. I felt embarrassed. It was a girls’ matter—how could I tell a male teacher? … Because he was a man, it was even harder to say.

(P001, student)

She also worried that classmates who did not experience menstrual pain might regard her as overly delicate or as exaggerating her symptoms. Remaining in class protected menstrual privacy and reduced the risk of negative judgement; it did not necessarily indicate that the pain was manageable.

In paid employment, seeking care could involve immediate financial loss as well as disclosure. P002 linked her decision not to consult to the limited benefit she expected from healthcare and the reduction in pay associated with taking leave:

It did not seem necessary. It was not a serious illness, and the doctor would just say it was normal anyway. Taking leave would also mean losing pay; it was not worth it.

(P002, factory worker)

For P002, anticipated reassurance and loss of income weighed in favour of continuing to work rather than seeking care. P017 faced a different disclosure problem and described dysmenorrhoea as stomach pain when requesting leave from a male employer:

The hardest part was having to ask for leave every time. My former boss was a man, and I was too embarrassed to say it was dysmenorrhoea, so I said I had stomach pain. Sometimes he would ask, ‘Why do you have stomach pain every month?’ I felt very embarrassed.

(P017, supermarket cashier)

Describing the symptom as ‘stomach pain’ allowed P017 to request leave without naming menstruation. However, her employer's questions about its monthly recurrence made these requests embarrassing.

P013 described having to take leave during both study and employment because pain prevented her from continuing her usual activities:

Every time I had my period, I had to ask for leave. Whether I was studying or working, I had to take leave. I did not want to move at all. The pain was so severe that I could not do anything.

(P013, self‐employed)

She explained that she sought care because the pain had become unbearable and she was unsure whether it was normal.

Disclosing menstrual pain could be difficult in both educational and workplace settings, as P001's and P017's accounts illustrated. For P002, taking time off to seek care also meant losing pay. Continued attendance at school or work could therefore reflect concerns about disclosure or the financial consequences of absence, even when pain disrupted daily activities.

Failure of previously effective self‐management provided another route into care. P016 had relied on ibuprofen but sought further assessment when medication no longer controlled the pain:

One time, the painkillers did not work at all. Usually one ibuprofen tablet could last half a day. That time I took two, but I was still rolling on the bed in pain, sweating all over. I almost called an ambulance. The next day my boyfriend insisted on taking me to the hospital.

(P016, administrative worker; participant‐reported diagnosis of endometriosis)

For P016, ineffective medication and her boyfriend's insistence prompted care‐seeking when private management was no longer sufficient. These accounts show how the effects of pain, the limits of self‐management and support from others could contribute to a decision to seek care.

### 3.4 Theme 4. Reproductive Expectations and Delayed Medicalisation

Participants recalled being told that menstrual pain would improve ‘after marriage’ or ‘after childbirth’. P002 and P007 described advice that linked getting married with having children. In these accounts, references to marriage were closely connected to expectations of childbirth and relief from pain. Such expectations could make seeking assessment seem less necessary.

The expectation that childbirth would relieve dysmenorrhoea was often transmitted through the experiences of mothers and other female relatives. P017, who had given birth and reported that her pain subsequently improved, described how her mother's experience influenced her earlier decisions:

I thought it was normal. Many girls in my class had pain, and some even had to ask for leave. My mother said she also had pain, and after giving birth to me she stopped having it. So I kept waiting for it to stop after I had a child.

(P017, supermarket cashier, parous)

P017's account shows how intergenerational experience made future improvement appear credible. Because childbirth was expected to change the pain, waiting could appear more appropriate than seeking assessment while she was younger.

P017 also described a culturally familiar bodily explanation in which childbirth was said to ‘open’ the uterus and allow menstrual blood to flow more freely:

People here say that after a woman gives birth, the uterus is ‘opened’, the menstrual blood flows through, and then it will not hurt anymore. My cousin was also like that. After giving birth, she stopped having pain.

(P017, supermarket cashier, parous)

P017 drew on her mother's and cousin's experiences, and later her own improvement, to explain why childbirth might relieve pain. P004 described a different experience: pain continued after marriage and childbirth, leading her to question the reassurance she had received. P017 also questioned whether childbirth explained her own improvement and cautioned against assuming that pain would resolve for everyone.

For other participants, reproductive concerns gave longstanding pain new significance. P007, a nurse, had previously normalised her dysmenorrhoea despite having healthcare knowledge. Difficulty conceiving prompted her to seek further attention:

It was after I got married. I had been trying to get pregnant for a long time and did not succeed, so I went for an examination. The doctor said it was gong han and that dysmenorrhoea was one of its signs. That was when I started to take it seriously. I am now taking Chinese medicine to regulate my body.

(P007, nurse)

P007's difficulty conceiving prompted her to seek care. The doctor's explanation in terms of gong han (‘cold uterus’) led her to reconsider pain she had previously regarded as normal. She reported taking Chinese medicine to regulate her body following the consultation.

P007 was explicit that, without concerns about conception, she might have continued to disregard the pain:

If it were not for trying to get pregnant, I probably still would not care about it now.

(P007, nurse)

Reproductive expectations therefore operated in different ways. Anticipated improvement after childbirth could postpone assessment, whereas difficulty conceiving gave pain new significance and provided a reason to seek care. Personal experiences could also lead participants to question earlier expectations of improvement.

### 3.5 Theme 5. Normal Tests, Uncertain Explanations and Delayed Re‐Entry Into Care

Healthcare contact did not always resolve participants’ concerns about menstrual pain. Responses to normal findings varied: some resumed self‐management, while others sought further assessment because they remained concerned about continuing pain.

P013 described seeking care at different hospitals because repeated reassurance did not resolve her concern about the cause of her pain:

I thought I needed to go to another hospital. … Sometimes I thought the hospital might not be able to find the problem, so I would go to another one. … Maybe there was some small abnormality they could not detect.

(P013, self‐employed)

P016 described a different response to her earlier consultations. Following ultrasound examinations at a township health centre, she accepted reassurance that the pain was not serious:

I had ultrasound examinations before. They all said the uterus and adnexa were normal. The doctor said, ‘It is nothing serious, primary dysmenorrhoea. Just keep warm.’ So I really thought it was nothing.

(P016, administrative worker; participant‐reported diagnosis of endometriosis)

She continued to manage the pain herself. Later, when ibuprofen no longer relieved it, her boyfriend took her to a municipal maternal and child health hospital. She recalled that another ultrasound was reported as normal, but the doctor recommended further investigation:

The doctor said, ‘A normal ultrasound does not mean there is no problem. Your dysmenorrhoea is so severe that I suspect endometriosis, and I suggest laparoscopy for diagnosis.’ I was stunned. I had not expected surgery.

(P016, administrative worker; participant‐reported diagnosis of endometriosis)

The later consultation offered a reason to investigate her continuing severe pain despite the normal scan. P016 reported undergoing laparoscopy in the year before the interview and receiving a diagnosis of endometriosis.

These accounts show that normal findings could be followed by different decisions about care. P013 remained concerned and sought assessment elsewhere, whereas P016 initially accepted reassurance and continued self‐management. Whether participants regarded the explanation as sufficient to account for their pain shaped their decisions about further care.

## 4 Discussion

### 4.1 Principal Findings

This study found that decisions about dysmenorrhoea care could change as women encountered family advice, tried treatments and sought clinical assessment. Pain that limited daily activities did not invariably prompt consultation. Some participants continued self‐management, while others sought care when symptoms worsened, treatment became ineffective or concerns about conception arose. After consultation, reassurance could support renewed self‐management, but continuing uncertainty could also prompt assessment elsewhere.

Healthcare candidacy draws attention to how people recognise a need for care, present that need and encounter professional judgements about it [22, 23]. The present analysis applies this perspective to recurrent menstrual pain. Family advice and peer comparisons shaped whether pain was considered to warrant care, while clinical explanations could reinforce or unsettle earlier understandings. These processes help explain how delays could occur both before an initial consultation and after women had already sought care. The need for assessment was reconsidered as symptoms, reproductive circumstances and experiences of treatment changed.

### 4.2 Negotiating Care‐Worthiness: Normalisation, Privacy and Functional Disruption

Family advice helped establish whether participants considered menstrual pain a reason to seek care. The importance of family communication is consistent with research among Chinese adolescent girls [18] and young women in Aotearoa New Zealand [37]. The present findings show how mothers’ explanations, grounded in their own experiences, could gain support through peer comparisons and clinical reassurance. Pain could thus be treated as ordinary across home, social and healthcare settings. Peer interactions could also encourage consultation or introduce another possible explanation for pain, giving women reasons to reconsider earlier advice.

Concerns about disclosure and absence help explain why disruption to daily life did not always result in seeking help. Students in Ireland have described prioritising productivity and concealing menstrual symptoms [38], while research in Spain has documented discomfort discussing menstruation in mixed‐gender settings [39]. In this study, difficulty disclosing symptoms to male authority figures was shared by students and working women. For those in paid work, taking leave could also mean losing income. Although menstrual symptoms can impair attendance, participation and concentration [40], continued attendance does not necessarily indicate manageable pain. Decisions about consultation also involved expectations of benefit, echoing concerns about healthcare reported among Ethiopian university students [41].

Similar difficulties extend to other aspects of women's health. Research with unmarried Chinese women has linked the stigma of gynaecological illness to decisions about disclosure and concealment [20]. A review of stigmatised urogenital and bowel symptoms in high‐income countries identified embarrassment, symptom normalisation and feeling dismissed by clinicians as barriers to seeking care [42]. In the present study, embarrassment could affect whether women disclosed menstrual pain and whether they felt able to identify it explicitly as a reason for seeking help or taking time away from work or study. From a candidacy perspective, recognising that pain disrupted daily life did not necessarily mean that women felt able to seek help for it.

### 4.3 Plural Explanatory Models: Warmth, Regulation and Reproductive Expectations

Participants used medicines to relieve pain while also trying to keep warm or nourish qi and blood. Similar use of medicines, heat and traditional herbs has been reported among Middle Eastern women living in Australia [43]. A review of Chinese populations found that traditional Chinese medicine was often viewed as complementary to Western medicine [44].

The present findings show how explanations involving cold, qi and blood could guide treatment choices alongside biomedical understandings of pain, while family beliefs also influenced whether treatment was continued.

For women who linked pain to cold, avoiding cold foods and keeping warm were ways of addressing what they believed caused their symptoms. Evidence supports topical heat for pain relief in primary dysmenorrhoea [45]. Within traditional Chinese medicine, cold‐induced blood stasis and qi and blood deficiency are among the patterns used to explain dysmenorrhoea and select treatment [16, 46]. One participant was told that she had insufficient qi and blood after a practitioner examined her pulse and tongue. She used prescribed herbs and soups prepared by her mother to nourish qi and blood. Both forms of care addressed what she understood as bodily weakness.

Concerns about medicines could alter these choices. Some participants limited analgesic use because they feared dependence, stomach problems or interference with menstrual flow. Family support for Chinese medicine could also change: one woman's relatives supplied a Chinese patent medicine, a ready‐made formulation, but later encouraged her to stop prescribed herbs because they feared kidney damage. Family influence therefore extended to decisions about stopping treatment, as well as choosing it.

One participant linked her pain to sensitivity to prostaglandins but also believed that cold foods could make it worse. Prostaglandins contribute to uterine contractions and reduced uterine blood flow in primary dysmenorrhoea; nonsteroidal anti‐inflammatory drugs relieve pain by inhibiting prostaglandin synthesis [47]. Her account combined a biomedical explanation with the belief that avoiding cold foods could help manage pain. This suggests that discussions about analgesics should address both how medicines work and women's beliefs about food and self‐care.

Expectations about childbirth also shaped the timing of consultation. In some accounts, advice that pain would improve ‘after marriage’ was closely linked with having children. Mothers’ and other relatives’ experiences made waiting for that improvement seem reasonable. Such expectations have some resonance with epidemiological evidence: an Australian longitudinal study found that severe period pain was less common among women who had had two births than among those who had not given birth or had had one birth [48]. Women in the present study, however, described both relief and continuing pain after childbirth, and some questioned earlier assurances. Difficulty conceiving could instead prompt consultation. For one woman, a clinician's explanation of gong han (‘cold uterus’) then led her to reconsider longstanding pain and begin Chinese medicine treatment. Expectations of future relief could postpone assessment, while fertility concerns brought pain to clinical attention.

### 4.4 Clinical Validation, Diagnostic Uncertainty and Re‐Entry Into Care

Clinical contact introduced professional explanations that women could accept or question. P016 initially accepted reassurance and continued self‐management, whereas P013 remained concerned and sought assessment elsewhere. These different responses suggest that the explanation accompanying a normal result mattered for decisions about further care.

Reviews of endometriosis research have identified normalisation and stigma as barriers to diagnosis [49] and documented long intervals between symptom onset and diagnosis [50]. In an Australian mixed methods study, participants commonly attributed diagnostic delay to dismissal and disbelief by healthcare professionals [51]. Imaging guidance makes clear that negative imaging does not exclude endometriosis, particularly superficial peritoneal disease [15, 52]. Clinical care also includes symptom management and follow‐up for primary dysmenorrhoea [14, 47]. The present findings show how a result described as normal could be understood as reassurance that continuing pain required no further attention. The findings therefore support explanations that link normal results to a plan for managing pain and reviewing symptoms, with further investigation where appropriate.

The ability to provide such explanations and follow‐up may also depend on the support and services available to clinicians. Studies of endometriosis care have identified needs for clearer diagnostic guidance, decision‐support tools and education among GPs in the Netherlands [53], while GPs in Aotearoa New Zealand described difficulties discussing menstruation and limited access to specialist services [54]. These studies indicate that clinical responses to persistent menstrual symptoms can be shaped by diagnostic support and access to referral. In Guizhou, primary care service scope has been shown to vary across facilities [55], while a study in Shuicheng County identified unequal distribution of rural doctors and basic medical equipment [56]. These local differences in service provision and resources may affect the capacity of primary care services to provide consistent assessment and follow‐up for persistent menstrual pain.

### 4.5 Implications for Culturally Responsive Education and Care

Three practical implications arise from the analysis. The first concerns education that addresses decisions women face when pain recurs. Participants described uncertainty about medicines and when to seek care. Some also suggested providing menstrual health information in schools or community settings. Practical information could cover appropriate analgesic use, the benefits and limitations of self‐management, and when symptoms or their effects on daily life warrant assessment. Discussions could address expectations of improvement after childbirth and include mothers and other family members whose advice influences care‐seeking. School‐based studies in East Asia and the Pacific have reported improvements in menstrual knowledge, although methodological limitations and limited assessment of broader outcomes remain [57].

The second implication concerns support in educational and workplace settings. Accounts of concealing menstrual symptoms or describing them as ‘stomach pain’ suggest a need for confidential ways to request rest, short‐term absence or temporary changes in tasks and schedules. An analysis of menstrual leave policy announcements identified potential benefits for reducing stigma, alongside concerns about reinforcing gender stereotypes and individual responsibility for managing menstruation [58]. Flexibility within general health and absence procedures could offer support without requiring women to name menstruation. Its design should consider both privacy and the financial consequences of taking leave.

The third implication concerns clinical communication. Clinicians could ask what women believe causes their pain, what treatments they use and how symptoms affect daily life. Discussing terms such as qi and blood or gong han may help clarify treatment expectations and patterns of medicine and home‐remedy use. When examination or imaging findings are normal, explanations should acknowledge continuing symptoms and include a clear plan for symptom management and review. This may help women distinguish reassurance about test results from the need to seek further assessment if pain persists or changes. In a Swedish cross‐sectional study, more validating and less invalidating communication was associated with better health‐related quality of life among women with endometriosis [59]. Healthcare professionals interviewed in Australia also emphasised identifying patients’ priorities and supporting their involvement in treatment decisions [60]. Future studies could examine whether discussing women's explanations of pain and agreeing a follow‐up plan support decisions about seeking further care.

### 4.6 Strengths and Limitations

Including women who had not sought care and women who had consulted clinicians allowed the analysis to address both initial care‐seeking and decisions about further assessment. Comparisons across educational and workplace accounts identified shared concerns about disclosure and the additional implications of lost income in paid work. Accounts of anticipated childbearing, difficulty conceiving and experiences after childbirth showed how reproductive circumstances could change the significance attached to pain. Analysis in Mandarin allowed the authors to examine culturally familiar terms in participants’ original accounts. Reflexive thematic analysis and multidisciplinary discussion supported consideration of alternative interpretations.

Most participants were Han Chinese, had rural backgrounds and had received tertiary education. Most were unmarried, and only three had given birth. Women with less formal education, women from minority ethnic groups and women with town/county backgrounds were less well represented. First‐hand accounts of experiences after childbirth were also limited. Recruitment through university, community and social‐network routes in Guiyang may also have favoured women who were more comfortable discussing menstruation.

Accounts of earlier menstrual experiences, family advice and consultations may have been influenced by later experiences. Some interview questions introduced possible explanations for pain or care‐seeking, which may have shaped the topics discussed and emphasised. Diagnoses and examination findings were reported by participants; medical records were not reviewed. Translation into English may also have lost some cultural nuance despite bilingual checking.

### 4.7 Conclusion

The accounts of young women in this study suggest that delayed care‐seeking for dysmenorrhoea was an evolving, socially negotiated process. Family advice, peer comparisons and culturally familiar explanations could support continued self‐management, even when pain disrupted daily life. Some participants sought or returned to care when symptoms disrupted daily functioning, existing management became ineffective, symptoms changed or reproductive concerns gave pain new significance. Normal findings could temporarily reassure participants or leave concerns unresolved and prompt further consultation. Healthcare candidacy helps explain how women's assessments of whether pain warranted care changed through everyday interactions and clinical encounters.

## Author Contributions

**Qingqing Zhu:** conceptualisation, methodology, investigation, writing – original draft, formal analysis, writing – review and editing, software, data curation. **Bentao Jia:** investigation, methodology, formal analysis, writing – review and editing, writing – original draft. **Qi Yu:** formal analysis, visualisation, writing – review and editing. **Mingqiang Wang:** investigation, resources, writing – review and editing, data curation. **Wei Xie:** investigation, writing – review and editing, resources. **Ling Qi:** formal analysis, validation, writing – review and editing. **Hongbo Chen:** writing – review and editing, project administration, resources. **Xiping Li:** conceptualisation, methodology, supervision, visualisation, funding acquisition, writing – review and editing. **Min Wang:** conceptualisation, methodology, supervision, writing – review and editing, funding acquisition.

## Ethics Statement

Ethical approval was granted by the Ethics Committee of the First Affiliated Hospital of Guizhou University of Traditional Chinese Medicine (Approval No. KY2024‐257A). All participants received information about the study and provided written or electronic informed consent before participation.

## Consent

All participants consented to the publication of anonymised interview data and quotations.

## Conflicts of Interest

The authors declare no conflicts of interest.

## Use of Artificial Intelligence Tools

ChatGPT was used solely to assist with spelling, grammar and general language editing during manuscript preparation. The authors reviewed and verified the final text and take full responsibility for the content of the manuscript.

## Data Availability

The data that support the findings of this study are available on request from the corresponding author. The data are not publicly available due to privacy or ethical restrictions.
